# Formulation of More Efficacious Curcumin Delivery Systems Using Colloid Science: Enhanced Solubility, Stability, and Bioavailability

**DOI:** 10.3390/molecules25122791

**Published:** 2020-06-17

**Authors:** Bingjing Zheng, David Julian McClements

**Affiliations:** 1 Biopolymers and Colloids Laboratory, Department of Food Science, University of Massachusetts Amherst, Amherst, MA 01003, USA; bingjingzhen@umass.edu; 2 Department of Food Science & Bioengineering, Zhejiang Gongshang University, 18 Xuezheng Street, Hangzhou 310018, China

**Keywords:** nanoliposomes, nanoemulsions, solid lipid nanoparticles, microgels, curcumin, functional foods

## Abstract

Curcumin is a bioactive constituent isolated from turmeric that has historically been used as a seasoning, pigment, and herbal medicine in food. Recently, it has become one of the most commonly studied nutraceuticals in the pharmaceutical, supplement, and food areas because of its myriad of potential health benefits. For instance, it is claimed to exhibit antioxidant, anti-inflammatory, antimicrobial, antiparasite, and anticancer activities when ingested as a drug, supplement, or food. Toxicity studies suggest that it is safe to consume, even at relatively high levels. Its broad-spectrum biological activities and low toxicity have meant that it has been widely explored as a nutraceutical ingredient for application in functional foods. However, there are several hurdles that formulators must overcome when incorporating curcumin into commercial products, such as its low water solubility (especially under acidic and neutral conditions), chemical instability (especially under neutral and alkaline conditions), rapid metabolism by enzymes in the human body, and limited bioavailability. As a result, only a small fraction of ingested curcumin is actually absorbed into the bloodstream. These hurdles can be at least partially overcome by using encapsulation technologies, which involve trapping the curcumin within small particles. Some of the most commonly used edible microparticles or nanoparticles utilized for this purpose are micelles, liposomes, emulsions, solid lipid particles, and biopolymer particles. Each of these encapsulation technologies has its own benefits and limitations for particular product applications and it is important to select the most appropriate one.

## 1. Introduction

Curcumin is a photochemical derived from turmeric, which is a perennial herb belonging to the Zingiberaceae family. Colloquially, turmeric is referred to as the “golden spice” because of its unique golden yellow color and earthy pungent flavor [1,2]. In the south and southeast Asian countries, turmeric has been used as a spice and pigment in food preparations for thousands of years. In addition, it has been widely used as an herbal medicine due to its perceived therapeutic benefits. Chemically, there are three major polyphenol substances that belong to the “curcuminoid” family: Curcumin (diferuloylmethane), demethoxycurcumin, and bisdemethoxycurcumin. Of these, the curcumin form is the most biologically active, and so it has been the focus in the development of pharmaceutical, supplement, and food products [3,4].

In functional food applications, curcumin can be considered as a natural ingredient that provides a distinctive color and flavor profile, as well as having potential health benefits [1,2,4]. Research in various fields has shown that the ingestion of curcumin may be beneficial to health due to its wide range of biological activities, including anti-inflammatory [5,6], antioxidant [6,7], antibacterial, antiviral, antifungal [8,9], antidiabetic, antitumor, and anticancer [3,10,11] activities. In cases where it is efficacious, curcumin may have advantages for the prevention or treatment of diseases because of its low cost, good safety profile, and lack of side effects. The research highlighting the potential benefits of curcumin has led to it being applied in a variety of commercial food and non-food products, including energy drinks, supplements, ointments, soaps, and cosmetics [12].

Pure curcumin is an orangey-yellow-colored crystalline material, which normally comes in a powdered form. Moreover, it is a chemically labile hydrophobic substance that has a low water solubility (particularly under acidic or neutral conditions, where it is fully protonated), poor chemical stability (especially under alkaline conditions), and low bioavailability (mainly due to low bioaccessibility and chemical transformation due to metabolic enzymes in the gastrointestinal tract). In addition, curcumin is susceptible to chemical degradation during storage, particularly when exposed to light, high temperatures, and alkaline conditions [13]. Although curcumin is relatively stable to chemical degradation under acidic conditions, it has a very low water solubility under these conditions, which can promote crystallization and sedimentation in aqueous delivery systems [14]. For this reason, it is important to develop effective approaches to overcome these hurdles so that curcumin can be successfully incorporated into pharmaceuticals, supplements, and functional food products.

One of the most effective means of protecting curcumin against chemical degradation, increasing its water dispersibility, and improving its bioavailability is to use modern encapsulation technologies [15,16]. These technologies involve incorporating the curcumin into edible nanoparticles or microparticles that can then be introduced into food or supplement products [17]. These colloidal particles are assembled from food-grade ingredients, such as surfactants, phospholipids, lipids, proteins, polysaccharides, and minerals, using either spontaneous or directed processes. Numerous kinds of colloidal particles can be employed for this purpose, including micellar aggregates, liposomes, emulsion droplets, solid lipid particles, and biopolymer particles [18].

## 2. Chemistry of Curcumin

**Figure 1:**
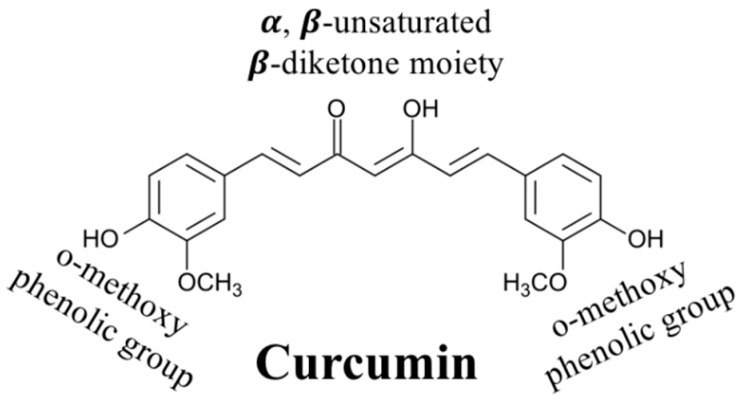
In general, the curcumin molecule consists of two phenolic groups held together by a diketone moiety.

**Figure 2:**
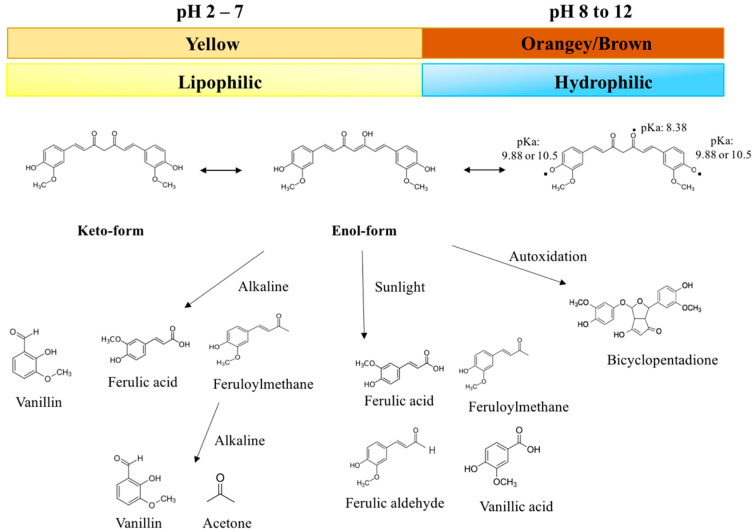
Curcumin chemically degrades when exposed to alkaline, sunlight, or autooxidation conditions, leading to a variety of reaction products with different molecular characteristics and bioactivities.

Curcumin (C_21_H_20_O_6_) is an asymmetric molecule with a molar mass of 368.38 g/mol (Figure 1). Structurally, it contains three main functional groups: Two aromatic ring systems containing o-methoxy phenolic groups, and one alpha beta-unsaturated beta-diketone moiety. In aqueous solutions, curcumin undergoes keto-enol tautomerism with its structure depending on pH: The keto form dominates under acidic and/or neutral conditions, while the enolate form dominates under alkaline conditions (Figure 2) [19,20,21]. The enol form is more chemically labile than the keto form, accounting for the poor chemical stability of curcumin in basic solutions [21].

## 3. Biological Activities of Curcumin

### 3.1. Antioxidant Activity

One of the main reasons that curcumin is used in many food formulations is due to its relatively strong antioxidant activity, which is believed to increase the shelf life of food products and protect cells from free radical-induced damage. Reactive oxygen species (ROS) generated inside the human body can promote the oxidation of lipids, proteins, and DNA molecules that place critical roles in normal cellular function. A number of chronic diseases have been linked to this phenomenon, including inflammation, cardiovascular disease, diabetes, and cancer [6,22,23]. Curcumin exhibits its antioxidant activity by acting as a free radical scavenger, singlet oxygen quencher, and chelating agent. For instance, it can donate a hydrogen atom from its β-diketone moiety to lipid alkyl or lipid peroxyl radicals, thereby reducing their activity [24,25]. In addition, it can chelate ferric (Fe^2+^) and ferrous (Fe^2+^) ions, which are known to be potent pro-oxidants. Some studies have also shown that it is highly effective at inhibiting the oxidation of emulsified lipids. For instance, in linoleic acid emulsions (droplet size not specified), a lower dose of curcumin (15 µg/mL or 20 mM) was required to inhibit lipid oxidation than butylated hydroxyanisole (123 mM), butylated hydroxytoluene (102 mM), tocopherol (51 mM), and trolox (90 mM) [7]. 

### 3.2. Anti-Inflammatory Activity

Curcumin is also widely used as a nutraceutical in functional foods because of its relatively strong anti-inflammatory activities. In particular, it has been reported that curcumin can suppress inflammatory response enzymes and transcription factors, such as TNF-a, IL-1, IL-6, IL8, IL12, monocyte chemoattractant protein (MCP)-1, cyclooxygenase-2 (COX-2), inducible nitric oxide synthase (iNOS), and lipoxygenase, thereby inhibiting the production of inflammatory cytokines [26,27]. The efficacy of curcumin for treating rheumatoid arthritis (a disease linked to inflammation of the joints) was compared to that of a widely used drug for this purpose (diclofenac sodium). After eight weeks, patients reported that curcumin formulation was more effective at reducing pain, swelling, and tenderness than the drug and that it exhibited less side effects. Moreover, the patients receiving the drug reported itching and swelling around their eyes, as well as dimness of vision [28]. Animal studies have also reported that curcumin reduced inflammation and bone erosion of collagen-induced arthritis (CIA) in rats after eight weeks of treatment (110 mg/kg) [29]. Other researchers have also claimed that the anti-inflammatory activity of curcumin is responsible for its ability to inhibit tumor formation and cancer.

### 3.3. Antimicrobial Effects

The antimicrobial activity of curcumin means that it has potential to inhibit food spoilage, thereby prolonging shelf life, as well as deactivating pathogenic organisms, thereby increasing food safety [30]. Moreover, the ingestion of curcumin-rich foods has the potential to treat or prevent some infectious diseases [31]. Several mechanisms of action have been proposed for the antimicrobial activity of curcumin, including its ability to increase the permeability of bacterial cell walls, inhibit microtubule formation, impair bacterial virulence factors, and interfere with key biochemical pathways [32]. For instance, studies have shown that there is an increase in cell membrane leakage for both Gram-negative (*S. aureus* and *E. faecalis*) and Gram-positive (*E. coli* and *P. aeruginosa*) bacteria after being treated with curcumin [33].

### 3.4. Anticancer

**Figure 3:**
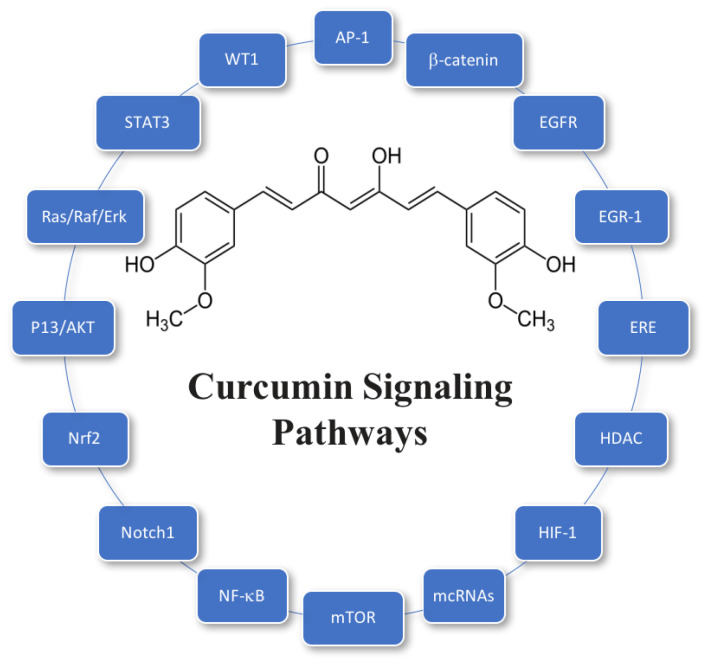
Proposed anticancer signaling pathways that curcumin can modulate. *Key*: AP-1, activating protein-1; EGR-1, early growth response protein-1; ERE, estrogen response element; HIF-1, hypoxia-in- ducible factor-1; mTOR, mammalian target of rapamycin; ncRNAs, non-coding RNAs; Nrf2, NF-E2-related factor 2; PI3K/AKT, phosphatidyli-nositide 3-kinases/protein kinase B; STAT3, signal transducer and activator of transcription 3; WT1: Wilms’ tumor 1 (adapted from Kunnumakkara, 2017).

Curcumin has also been reported to have the ability to inhibit the growth of cancer cells by suppression of angiogenesis and induction of apoptosis [34,35]. In vitro and in vivo studies suggest that curcumin may be able to downregulate cell growth and proliferation in various types of cancer cells, including prostate, breast, and colon cancers [34]. In the case of prostate cancer, curcumin downregulated cancer cell proliferation by attacking epidermal growth factor receptors [36]. Curcumin has also been shown to suppress cell motility and metastasis by inhibition of bone metastatic LNCaP-derivative C4-2B prostate cancer cells [36,37]. In the case of breast cancer, curcumin has been reported to mediate breast cancer cell apoptosis via suppression of NFκB, cyclinD, and MMP-1 expression [38]. In the case of colon cancer, curcumin had been reported to reduce miR-21 promoter activity and expression by inhibiting HCT116 cells and Rko cells in the G_2_/M phase, which regulates progression and metastasis of cancer cells [39,40]. In general, studies have identified multiple signaling pathways that curcumin can modulate to produce anticancer effects, which often involve the targeting of multiple key components within these pathways [41]. A summary of a number of molecular targets of curcumin associated with its anticancer activity is given in Figure 3. It should be noted that other molecular targets have been identified for other diseases that curcumin can prevent or treat [42].

## 4. Potential Toxicity

The potential toxicity and side effects of ingesting curcumin have been investigated for decades using both animal and human models. A human feeding study reported no toxicity when up to 8 g of curcumin were ingested every day for three months; however, some of the test subjects did report minor side effects, such as diarrhea or nausea [43]. Another human study reported only minor side effects (diarrhea, rash, headache, and yellow stool) in one subject when they were fed relatively high levels (1–12 g per day) of curcumin for prolonged periods [44]. After reviewing the available evidence, the United States Food and Drug Admission considers curcumin to be generally regarded as safe [45]. The United Nations and World Health Organization Expert Committee on Food Additives, as well as the European Food Safety Authority, allow a relatively high daily intake of curcumin: 0 to 3 mg/kg body weight/day [46,47], which corresponds to up to about 210 mg day for an average person. This level is well above that reported to have beneficial health effects in human feeding studies [48]. However, a recent cell culture model showed that curcumin could exhibit beneficial anticancer effects at lower doses but exhibit toxicity at relatively high doses [49]. These results suggest that it is important to consider both the dose and bioavailability of the curcumin in specific formulations.

## 5. Factors Affecting Curcumin’s Application 

In this section, some of the main challenges that need to be addressed when formulating curcumin-based functional foods are discussed.

### 5.1. Solubility

At room temperature, pure curcumin is a crystalline material with a melting point around 183 °C. In addition, it is a predominantly hydrophobic substance due to the non-polar regions in the aliphatic bridge, aromatic rings, and methyl groups (Figure 1) [50]. Nevertheless, it does have three hydroxyl groups, which become deprotonated at sufficiently high pH values, thereby giving it a negative charge (Figure 2). Consequently, curcumin is a predominantly hydrophobic molecule with low water solubility under acidic and neutral conditions (where the hydroxyl groups are protonated), but a hydrophilic molecule with a relatively high water solubility under alkaline conditions (where the hydroxyl groups are deprotonated) [51]. In particular, the solubility of curcumin increases as the solution pH is raised around and above the pK_a_ values of the three hydroxyl groups (8.38, 9.88, and 10.51), which are located in the enolic (pK_a_:8.38) and phenolic regions (pK_a_: 9.88 and 10.51) of the molecule (Figure 1 and Figure 2) [52]. For example, below about pH 8, the net charge = 0, the log D = 4.1, and the water solubility are very low (around 24 mg mL^−1^ or 0.0024%). Conversely, at pH ≥ 12.0, the net charge = −3, the log D = −2.0, and the water solubility are very high (> 3 g mL^−1^). As well as leading to an increase in water solubility under alkaline conditions, deprotonation of these hydroxyl groups also promotes a color change and an increase in chemical instability (see the following sections). In most foods, the pH ranges from about 2 to 8, so that the curcumin is a predominantly hydrophobic molecule with low water solubility. As a result, it typically needs to be dissolved in some form of hydrophobic substance before it can be incorporated into aqueous-based foods; otherwise, it will be in a crystalline form. Having said that, the pH-dependence of the water-solubility of curcumin can be utilized in the formation of colloidal forms of curcumin, e.g., in the pH-shift method (see later).

### 5.2. pH-Induced Color Changes

The color of curcumin solutions depends on the protonation state of the three hydroxyl groups and therefore changes with pH (Figure 2). From pH 2 to 7, all of the hydroxyl groups are protonated, and the curcumin appears golden yellow, which is the case in most foods. From pH 7 to 8.5, the enolic hydroxyl group becomes progressively deprotonated, causing the curcumin to change to a more brownish-orangey color. At still higher pH values, the other two phenolic hydroxyl groups become deprotonated, causing the curcumin to have a more reddish color [4,13,53]. The chemical state of curcumin under specific solution conditions can therefore be elucidated by measuring the UV-visible absorption spectrum [4]. It should be noted that curcumin chemically degrades under alkaline conditions, which causes changes in its color. 

### 5.3. Chemical Degradation

#### 5.3.1. Alkaline Degradation

As mentioned earlier, the water solubility of curcumin increases under alkaline conditions, which contributes to an increased rate of chemical decomposition. At pH values around and above the pK_a_ values of its hydroxyl groups, curcumin undergoes rapid hydrolytic degradation, which has been attributed to cleavage of the α, β-unsaturated β-diketone moiety. As a result, the original curcumin molecule is transformed into trans-6-(4′-hydroxy-3′-methoxyphenyl)-2,4-dioxo-5-hexanal, which itself undergoes cleavage reactions to form ferulic acid, feruloylmethane, and vanillin [4,13,53,54] (Figure 2). The color of curcumin fades due to this alkaline degradation reaction. In phosphate buffer solutions, it has been reported that around 90% of curcumin degraded within 15 min of incubation at neutral or alkali conditions, but the molecule was relatively resistant to degradation under acidic conditions [14,55]. The possible degradation of curcumin under neutral and alkaline conditions must therefore be considered when developing curcumin-enriched functional foods.

#### 5.3.2. Photodegradation

Curcumin (crystalline or solubilized) also undergoes chemical degradation when exposed to light, which promotes color fading [56,57]. The photodegradation of curcumin is also initiated at the α, β-unsaturated β-diketone moiety and leads to a variety of reaction products, including p-hydroxybenzaldehyde, vanillin, vanilic acid, ferulic aldehyde, and ferulic acid (Figure 2) [56]. Typically, the crystalline form of curcumin is more stable to photodegradation than the solubilized form, which may be because a higher fraction of the light waves is able to penetrate into a clear solution. Certain reaction products (e.g., vanillin and ferulic acid) have been reported to have some antioxidant and anticancer activities, but they are less potent than the curcumin molecule itself [58,59,60].

#### 5.3.3. Autoxidation

Curcumin may also chemically degrade due to autoxidation reactions that occur spontaneously in aqueous solutions via a radical chain reaction [61,62]. Initially, free radicals in the surrounding solution initiate autoxidation of the phenolic hydroxyls on the curcumin molecule, which results in the formation of an unstable intermediate that breaks down through a series of reactions to form bicyclopentadione [61,63]. This reaction product has been shown to exhibit some anticancer activity but less than that of curcumin itself [63,64].

### 5.4. Bioavailability

**Figure 4:**
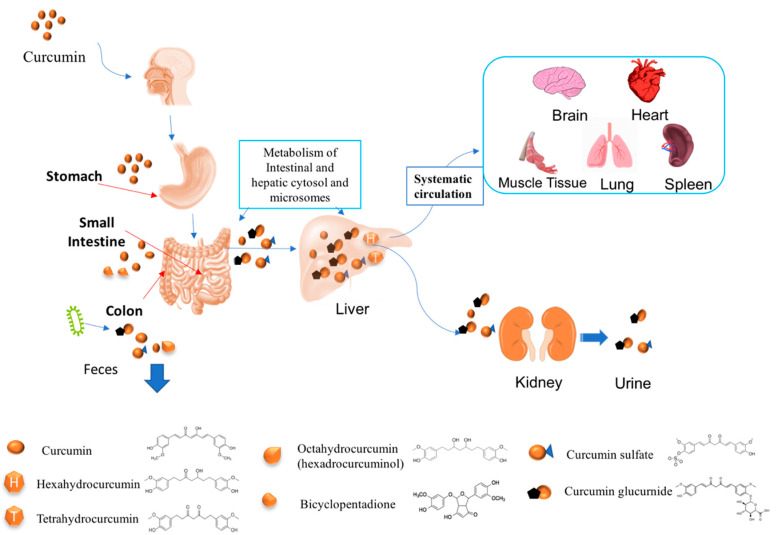
Curcumin undergoes chemical degradation due to metabolism as it passes through the human gut and body. The metabolites formed have different bioactivities to the parent molecule.

In this section, some of the main factors limiting the bioavailability of curcumin are highlighted (Figure 4).

#### 5.4.1. Bioaccessibility, Chemical Transformation, and Absorption

The high melting point and low water solubility of curcumin under acidic and neutral conditions mean that pure curcumin crystals typically have a low bioaccessibility. In other words, the crystals do not readily dissolve in the aqueous gastrointestinal fluids, which reduces their ability to be transported through the mucus layer and be absorbed by the epithelium cells [34,65]. The bioavailability of curcumin may also be limited due to chemical transformation within the gastrointestinal tract. Curcumin is resistant to degradation under acidic environments, and should therefore remain stable in the stomach. Conversely, it is prone to alkaline degradation under neutral or basic conditions and so may be unstable in the small intestine and colon. Some studies have shown that curcumin may also undergo autoxidation under physiological pH conditions [3]. Despite these potential degradation mechanisms, a rat study reported that around 90% of ingested curcumin remained in the gastrointestinal tract (GIT) after exposure to the stomach and small intestine conditions for 30 min, suggesting that its degradation was relatively slow within the gut [66]. The bioaccessibility and chemical stability of curcumin can be increased by encapsulating it within a lipid phase, such as a bulk or emulsified oil [50,67]. Trapping the curcumin within a lipid phase protects it from chemical degradation by physically isolating it from reactants in the aqueous gastrointestinal fluids. Moreover, the utilization of a digestible lipid phase (such as a triglyceride) leads to the production of lipid digestion products (fatty acids and monoglycerides) that are incorporated into mixed micelles. These mixed micelles can then solubilize the curcumin within their hydrophobic interiors, thereby enhancing its bioaccessibility. Moreover, they can transport the hydrophobic curcumin molecules to the epithelium cells where they can be absorbed. After absorption, the curcumin may be metabolized within the epithelium cells and/or expelled back into the intestinal lumen due to the presence of efflux transporters within the cell membranes [68,69]. Some substances within foods, such as piperine in black pepper and certain catechins in green tea, are able to inhibit these efflux transporters, therefore increasing the amount of curcumin remaining in the body [70,71]. This gives food formulators an approach to increase the potential bioactivity of curcumin using common food ingredients that act as efflux inhibitors [72].

#### 5.4.2. Metabolism

One of the main reasons for the poor oral bioavailability of curcumin is its rapid metabolism by metabolic enzymes inside the gut, as well as after absorption (particularly in the liver), leading to the formation of a variety of metabolites with different biological activities to the parent molecule [65]. The human intestine and liver contain phenol sulfotransferase isoenzymes that convert curcumin into curcumin sulfates, as well as glucuronidases that convert curcumin into curcumin glucuronides [73]. In addition, a number of other metabolites may be formed, including bicyclopentadione, dihydrocurcumin, tetrahydrocurcumin (THC), hexahydrocurcumin (HHC), octahydrocurcumin (OHC), hexahydrocurcuminol, dihydroferulic acid, and ferulic acid [72,74]. The majority of the curcumin glucuronides, curcumin sulfates, and other metabolites are fairly water soluble and so are quickly excreted from the body via the urine and feces [75,76]

A number of the metabolites of curcumin have been found to exhibit some biological activity. THC has been reported to have stronger anti-inflammatory, antidiabetic, and antihyperlipidemic activity than curcumin, as well as a similar antioxidant activity [22,58,77]. HHC has been reported to have similar or better antioxidant, anti-inflammatory, anticancer, and cardiovascular protective activities as curcumin [78,79,80]. OHC has also been reported to have superior anticancer properties than curcumin, by being more effective at suppressing tumor growth and inducing cancer cell apoptosis [81,82]. In contrast, curcumin glucuronide has been reported to have a lower absorption and anticancer activity than curcumin [83]. Curcumin glucuronides and curcumin sulfates have also been reported to be less bioactive than other metabolites, as well as the parent molecule [84]. 

#### 5.4.3. Tissue Distribution

After absorption, curcumin enters the bloodstream and is rapidly distributed throughout the body, resulting in it being located in many tissues at detectable levels, including the liver, kidney, colon, brain, heart, lung, and spleen [66,72,85,86,87]. For instance, a rat study reported that a small amount of curcumin was found in the liver and kidney soon after oral administration, while about 38% was present in the large intestine after 24 h [66]. The maximum curcumin concentration was detected in the liver (around 45 µg/whole tissue) and kidney (6 µg/whole tissue) after 3 h. The amount of curcumin in the kidney has been reported to decline after about 24 h, while that in the liver remains fairly constant for up to 4 days [86]. A rat feeding study reported that different levels of curcumin were detected in different tissues after oral administration: Liver (70 nmol/mL), kidney (78 µmol/mL), brain (3 nmol/mL), lung (15 nmol/mL), heart (9 nmol/mL), and muscle tissue (8 nmol/mL) [85]. These results suggest that curcumin has the potential to work in various tissues within the human body, which means that it may be able to treat a variety of different disease conditions. 

#### 5.4.4. Elimination

Curcumin is eliminated from the body more through the feces than through the urine, which may be due to its relatively low water solubility [87]. An animal study suggested that about 34% of curcumin was excreted through the feces, while less than 0.2% was secreted in the urine [86]. Nevertheless, curcumin metabolites (such as glucuronides and sulfates), which are much more water soluble than the parent molecule, tend to be excreted through both the urine and feces [75,76].

#### 5.4.5. Pharmacokinetics

Pharmacokinetic studies have been used to study the levels of curcumin in the bloodstream of animals and humans after oral ingestion. These studies typically show that the fraction of curcumin reaching the bloodstream in an intact form is very low. For instance, in an animal study, it was reported that there was only about 0.22 µg/mL of curcumin in blood samples taken an hour after oral administration of 1.0 g curcumin per kg body weight, with this concentration declining over the following 6 h [88]. In a human trial, only around 11 nmol/L curcumin was detected in blood plasma collected an hour after oral administration of 3.6 g of curcumin [76]. In human feeding studies, even a relatively high intake of curcumin (8 g per day) led to a relatively low serum level (2 µm/mL) in blood samples collected an hour or two after consumption [43]. These studies indicate that only a very small fraction of ingested curcumin actually gets into the systemic circulation in humans, which may limit its potential biological activity.

## 6. Strategies to Overcome the Challenges of Curcumin

Potential strategies to improve the solubility/dispersibility, stability, and bioavailability of curcumin are highlighted in this section.

### 6.1. Methods to Enhance Solubility/Dispersibility of Curcumin

The solubility of curcumin in both oil and water phases is important when developing effective formulations to encapsulate and deliver it. At room temperature, pure curcumin is typically in a powdered crystalline form. Consequently, it must be dissolved or dispersed within an appropriate solvent before it can be incorporated into a suitable food format. In this section, a number of approaches for introducing curcumin into solvents are highlighted.

#### 6.1.1. Direct Dissolution

Curcumin has a relatively low water solubility (under neutral or acidic conditions), but it can be directly dissolved within oils and some organic solvents due to its lipophilic nature. It should also be noted that curcumin tends to exist in the keto-form in water (under most pH conditions found in foods) but in the enolic form in oils and organic solvents (Figure 3). Some of the most common organic solvents used to directly solubilize curcumin are ethanol, methanol, chloroform, acetone, and dimethoxy sulfoxide [4]. These solvents are often used to dissolve curcumin prior to creating colloidal delivery systems. For instance, ethanol is often used to solubilize both curcumin and particle-forming materials, such as surfactants, phospholipids, hydrophobic proteins, or hydrophobic polysaccharides. Colloidal particles are then formed using an antisolvent precipitation method by injecting the ethanol mixture into water [89,90]. When the hydrophobic curcumin and particle-forming materials come into contact with water, curcumin-loaded particles are spontaneously formed. Curcumin has been loaded into zein nanoparticles using this method [91]. One disadvantage of using organic solvents for this purpose is that they may be environmentally unfriendly and additional costs are associated with removing and analyzing them in the final formulation [92,93]. This problem can be overcome by using supercritical fluids (such as supercritical carbon dioxide) to dissolve the curcumin [94,95]. Alternatively, alkaline water can be used as a solvent, rather than an organic fluid.

As mentioned earlier, the solubility of curcumin increases substantially when the solution is raised above about pH 9 because the molecule changes from hydrophobic to hydrophilic (Figure 2). Consequently, curcumin can be solubilized in highly alkaline solutions. Curcumin-loaded colloidal particles can then be formed using a pH-shift method that involves injecting the alkaline curcumin solution into an acidified aqueous colloidal suspension. The pH decreases when these two systems are mixed together, which causes the curcumin to become more hydrophobic and move into the non-polar regions within the colloidal particles. This approach has been used to encapsulate curcumin into surface micelles [96], solid lipid particles, liposomes [97], emulsions, protein nanoparticles [98,99], and oil bodies [99,100]. The curcumin should only be kept for a relatively short time under highly alkaline conditions to avoid its degradation. 

#### 6.1.2. Mechanical Action

The dissolution of crystalline curcumin in solvents can be increased by applying mechanical forces, such as stirring and sonication. Sonication is particularly suitable for this purpose because it generates intense fluctuating pressure waves that induce acoustic cavitation, leading to efficient mixing and dissolution [101,102].

#### 6.1.3. Heating

The solubility of crystalline materials in solvents usually increases as the temperature is raised. Consequently, it is possible to solubilize a higher concentration of curcumin by heating the system. This approach has been used to facilitate the dissolution of powdered curcumin into bulk oils, prior to emulsion formation [103]. It has also been used to increase the dissolution of powdered curcumin into pre-existing emulsions [104].

#### 6.1.4. Encapsulation Technologies

One of the most common approaches to improve the water dispersibility of curcumin is to incorporate it within colloidal particles that have a hydrophobic interior but a hydrophilic exterior, such as micelles, microemulsions, emulsions, or hydrophobic biopolymer particles. Numerous kinds of colloidal particles that can be used for this purpose are covered in more detail in Section 6.3.

### 6.2. Methods to Enhance Stability of Curcumin

As mentioned earlier, curcumin is susceptible to chemical transformation when exposed to certain conditions, such as alkaline pH, light, elevated temperatures, transition metals, and metabolic enzymes. For this reason, it is necessary to develop effective strategies to protect it from chemical degradation so that it can reach the target organs in an active state.

#### 6.2.1. Antioxidant Technologies

As mentioned in Section 3.1, curcumin can be used in foods as a natural antioxidant. In these cases, the curcumin is usually chemically transformed, which may alter its biological activity. In many applications, it is desirable to prevent the chemical degradation of curcumin to maintain its beneficial biological activities after ingestion. The chemical degradation of curcumin can be inhibited by adding synthetic or natural antioxidants [105]. These authors reported that co-administration of curcumin with certain antioxidants decreased the degradation rate and increased the amount absorbed after oral administration to rats. Specifically, a number of food-grade redox-active antioxidants were shown to greatly improve curcumin stability, including ascorbic acid, gallic acid, caffeic acid, rosmarinic acid, tert-butylhydroquinone, and Trolox. The addition of antioxidants has also been shown to enhance the stability of curcumin encapsulated within oil-in-water emulsions [106]. Hydrophilic and amphiphilic antioxidants were found to be more effective than lipophilic ones in this study, which may be because the chemical degradation of curcumin occurs more rapidly in the water phase. Overall, the authors reported the following order of efficacy for different antioxidants: Trolox ≈ ascorbic acid > ascorbyl palmitate >> control > alpha-tocopherol. These results suggest that the stability of curcumin in food formulations can be improved by adding appropriate antioxidants. 

#### 6.2.2. Encapsulation Technologies

The chemical degradation of curcumin occurs faster when it is surrounded by water than oil, because substances that accelerate the degradation reaction (such as hydroxyl ions) are mainly located within the aqueous phase [14]. Consequently, the stability of curcumin to chemical degradation can be improved by encapsulating it within a hydrophobic phase, which may be a bulk phase (such as pure oil) or colloidal particles (such as oil droplets, solid fat particles, or hydrophobic protein particles) [14,107,108]. Interestingly, the chemical stability of curcumin increases as the size of these hydrophobic colloidal particles increases, because then there is a slower exchange of curcumin molecules between the particles and the surrounding aqueous phase [109]. Encapsulation technologies that utilize colloidal particles to improve the chemical stability and bioavailability of curcumin in aqueous-based systems are discussed in Section 6.3.

#### 6.2.3. Controlling Environmental Conditions

Curcumin is known to degrade faster when exposed to light [110], high temperatures [14], high oxygen levels [111], and alkaline environments [14]. It is therefore possible to improve its stability by controlling the solution, environmental, and/or packaging conditions. For instance, the chemical stability of curcumin can be improved by incorporating it into acidic products (pH < 7) that are stored at low temperatures in the dark, e.g., fruit juices, dressings, or some nutritional beverages [50,67]. Alternatively, it may be possible to exclude light and oxygen by using appropriate packaging procedures and materials, thereby further enhancing the stability of curcumin-based products. 

### 6.3. Methods to Enhance the Bioavailability of Curcumin

The bioavailability of curcumin can be enhanced by retarding its metabolism, increasing its bioaccessibility, and/or promoting its absorption. The enzymes that metabolize curcumin are located within the aqueous phase or within the cell membranes inside the human body. Consequently, the metabolism of curcumin can therefore be inhibited by trapping it inside hydrophobic phases that isolate it from the enzymes, such as those inside micellar, liposomal, microemulsion, emulsion, solid fat, or biopolymer particles. The bioaccessibility of curcumin can be increased by enhancing the amount that is solubilized within the mixed micelles present in the small intestine. This may be achieved by including surfactants, phospholipids, fatty acids, or monoglycerides within the curcumin-loaded carrier particles, as these surface-active substances, can become incorporated into the mixed micelles and increase their solubilization capacity. Alternatively, this can be achieved by including digestible lipids within the curcumin-loaded carrier particles, such as triglycerides or diglycerides. These lipids are then converted into monoglycerides and fatty acids by lipase in the human gut, thereby generating surface-active materials that can be incorporated into the mixed micelles and enhance their solubilization capacity. Finally, substances that increase the permeability of the epithelium cell membranes or that block efflux transporters can be incorporated into the curcumin-loaded carrier particles. For instance, some surfactants, fatty acids, biopolymers, and phytochemicals have been shown to increase the permeability of epithelium cells, thereby leading to enhanced absorption of hydrophobic bioactives [112,113,114]. As mentioned previously, certain kinds of food components, including piperine in black pepper and some catechins in green tea, can inhibit efflux transporters, thereby increasing the amount of curcumin absorbed by the body [70,71]. 

## 7. Colloidal Delivery Systems

**Figure 5:**
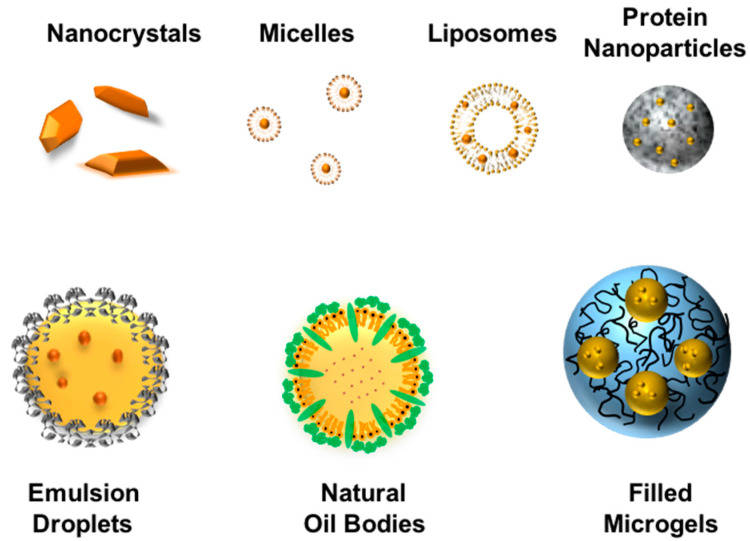
Different kinds of colloidal delivery system can be used to encapsulate curcumin, thereby improving its stability and bioaccessibility (not drawn to scale).

Colloidal delivery systems have been widely explored for their ability to increase the bioavailability of polyphenols, like curcumin [18]. Numerous kinds of colloidal delivery systems have been shown to be suitable for this purpose, including micelles, microemulsions, emulsions, solid lipid nanoparticles, protein nanoparticles, and biopolymer microgels. The curcumin-loaded colloidal particles can then be incorporated into functional food and beverage systems, or converted into a powdered form and used in supplements. Many factors impact the selection of a colloidal delivery system for a particular application: Particle characteristics (composition, size, shape, morphology, charge, digestibility, stability), loading properties (encapsulation efficiency, loading capacity, retention efficiency), physicochemical properties (optical properties, rheology, shelf-life), sensory attributes (flavor profile, mouthfeel), economics (ingredient and manufacturing costs), regulations, and label friendliness (all natural, vegan, Kosher) [115]. Excipient foods can be used as an alternative strategy to delivery systems for enhancing the bioavailability of curcumin. An alternative strategy is to use excipient foods to enhance the bioavailability of curcumin. An excipient is a food that contains no bioactive components itself but breaks down in the human gut to form an environment that enhances the bioaccessibility, stability, or absorption of any curcumin co-ingested with it. Emulsified foods containing digestible lipids are particularly suitable for this purpose, including milk, creams, dressings, sauces, or yogurts [116]. A schematic diagram of different colloidal delivery systems that can be used to encapsulate curcumin is shown in Figure 5.

### 7.1. Micelles

Micelles have been widely used to improve the solubility and bioavailability of hydrophobic drug and nutraceuticals [117,118]. They are spontaneously formed when surfactants are dispersed in water above their critical micelle concentrations. Consequently, they can often be fabricated using very simple processing methods, such as heating and/or mixing of the bioactive, surfactant, and water together. After assembly, the hydrophobic tails congregate together in the interior of the micelles, while the hydrophilic head groups point outwards towards the water. Micelles are typically formed from synthetic surfactants, but they can also be formed from certain kinds of natural surfactants, such as casein [119,120]. Curcumin can be solubilized within the hydrophobic interior of the micelles by adding it before or after micelle formation. Typically, micelles are relatively thermodynamically stable colloidal dispersions containing relatively small (typically 5 to 20 nm) particles. As a result, they tend to be optically transparent because the particles are so small that they do not scatter light waves strongly. The viscosities of micellar systems depend on the surfactant concentration and micelle structure. At low concentrations, they tend to be fluids with low viscosities, whereas at higher concentrations, they tend to be semi-solids with high viscosities or gel-like textures. The rheological properties of micellar systems depend strongly on the surfactant type and environmental conditions because these factors influence the size, shape, interactions, and dynamics of the colloidal structures formed [121]. Micelles enhance the bioavailability of hydrophobic compounds (like curcumin) by increasing their bioaccessibility in the gastrointestinal fluids, as well as possibly increasing the permeability of the epithelium cells [122]. 

Synthetic food-grade surfactants, such as Tweens, have been widely used to solubilize curcumin [123]. The solubilization of curcumin has been reported to increase with increasing chain length and decreasing the unsaturation of the surfactant tail groups [123]. Natural surfactants, such as casein, have also been used to solubilize curcumin within aqueous solutions [124], which may be more suitable for certain food applications. The pharmacokinetics of curcumin-fortified Tween 80 micelles have been tested in a human feeding study. The curcumin–micelle formulation was found to have a 185-fold higher area under the curve than free curcumin, without exhibiting any adverse side-effects [125]. 

### 7.2. Liposomes

Liposomes are self-assembled spherical particles consisting of one or more phospholipid bilayers [93,126]. They therefore have a structure that is somewhat similar to biological cell wall membranes. A phospholipid molecule consists of a hydrophilic head group and a hydrophobic tail group consisting of two fatty acid chains. In a single bilayer, the phospholipid molecules are organized tail to tail, therefore forming a thin hydrophobic domain, while the polar head groups face outwards. Liposomes containing multiple bilayers often have an onion-like structure with concentric rings of individual bilayers. Liposomes therefore have polar, non-polar, and amphiphilic regions inside the same colloidal particle, which can be useful for encapsulating one or more bioactive agents with different polarities within a single delivery system. Liposomes vary greatly in dimensions, ranging from around 25 nm to 25 µm depending on the formulation and fabrication method used. Curcumin-loaded liposomes typically have an orangey-yellow clear or slightly turbid look depending on the particle size [91]. 

Liposomes can be prepared using various methods, including passive-loading, active-loading, mechanical-dispersion, solvent-dispersion, and detergent-removal methods [126]. The preparation method used affects the nature of the liposomes formed, as well as the loading capacity of the curcumin. The characteristics of curcumin-loaded liposomes prepared using three different methods have been compared:Thin film, ethanol injection, and pH-driven methods [97]. The initial diameters of the liposomes decreased in the following trend: Thin film (452 nm) > pH-driven (217 nm) > ethanol injection (115 nm). The initial encapsulation efficiency of the liposomes for curcumin decreased as follows: Thin film (78%) > pH-driven (66%) > ethanol injection (39%). The physical and chemical stability of the curcumin-loaded liposomes also depended on the fabrication methods. After 30 days of storage in the dark (4 °C), the mean particle diameters increased to 1650, 234, and 153 nm for the thin film, pH-driven, and ethanol injection methods, while the curcumin concentration decreased by 50%, 2%, and 2%, respectively. The effect of the ionic strength (0.1 to 1 M NaCl) on the stability of the liposomes was also assessed. Overall, the salt stability of the emulsions decreased as their initial particle size increased. In particular, the liposomes prepared by ethanol injection (which had the smallest initial size) were stable to salt addition, with little change in their appearance at any salt level after storage. Conversely, an increase in turbidity and precipitation were observed in the other liposome suspensions at high salt levels. Overall, the results suggest that controlling the liposome size is important for maintaining good product stability [97]. In another study, it was shown that curcumin-loaded liposomes could inhibit the chemical degradation of curcumin, especially when exposed to transition metal ions (Fe^3+^, Fe^2+^, Al^3+^, and Cu^2+^) [127]. 

The phospholipid source may also influence the physicochemical characteristics of curcumin-loaded liposomes. One study compared the properties of curcumin-loaded liposomes produced using a thin film/ultrasonic dispersion method that were fabricated from either milk fat globule membrane (MFGM) or soybean lecithin [128]. There were differences in the encapsulation efficiency, mean particle diameter, and ζ-potential of the liposomes depending on the lecithin type: EE = 74%, d = 212 nm ζ = +7.6 mV for MFGM; EE = 63%, d = 471 nm and ζ = −48.6 mV for soybean lecithin. The encapsulation of the curcumin was also shown to protect it from degradation when exposed to alkaline conditions, Fe^3+^, light, heating, oxygen, and relative humidity, with the MFGM liposomes giving slightly better protection than the soybean lecithin ones [128]. Other studies have also shown that the lecithin type influences the encapsulation efficiency and stability of curcumin in liposomes [129]. 

The impact of liposome encapsulation on the bioavailability of curcumin has been studied using both in vitro and in vivo experiences. Interestingly, an in vitro gastrointestinal tract study showed that curcumin in larger liposomes (200 or 450 nm) had a higher bioaccessibility than that in smaller ones (114 nm) [97]. An animal feeding study was used to investigate the oral bioavailability of the two curcumin-loaded liposome formulations: Flexible liposomes (FLs) and silica-coated flexible liposomes (SLs) [130]. Pure curcumin suspended in water was used as a control. The curcumin concentrations within the blood plasma of the animals were measured up to 12 h after oral administration. The maximum curcumin concentration occurred after 45 min for both the FL formulation and the control, with values of 129 and 71 ng/L, respectively. Conversely, the maximum curcumin concentration occurred after 3 h for the SL formulation, with a value of 447 ng/L. Moreover, no curcumin was detected in the plasma of the animals after 4 h for the control, while around 20 ng/L still remained after 12 h for the encapsulated curcumin. Overall, both the SL (7.8-fold) and FL (2.4-fold) formulations gave a higher total amount of curcumin within the blood compared to free curcumin [130]. Thus, both liposome formulations were effective at increasing curcumin bioavailability. 

Other researchers have also used an animal model (mice) to study the impact of liposomal encapsulation of curcumin on its bioavailability [129]. The maximum plasma concentration and time to reach this value depended on the formulation used: Free curcumin (C_max_ = 64 µg/L; T_max_ = 120 min); curcumin-loaded liposomes (C_max_ = 319 µg/L; T_max_ = 30 min); and free curcumin + liposomes (C_max_ = 78 µg/L; T_max_ = 120 min). These results show that the bioavailability can again be increased by delivering curcumin in the form of liposomes. Importantly, liposomal curcumin also exhibited higher plasma antioxidation activity than the other two curcumin formulations [129], which can be attributed to its higher plasma level. Curcumin-loaded liposomes have been shown to exhibit the same cellular antioxidant activity as free curcumin when exposed to Caco-2 cells for 2 h [127], which shows that encapsulation did not reduce the bioactivity of this nutraceutical.

### 7.3. Microemulsions

In general, microemulsions are thermodynamically stable isotropic colloidal dispersions formed from oil, water, and surfactant. Oil-in-water microemulsions contain small colloidal particles (typically 5 to 50 nm) comprised of oil and surfactant, which have a hydrophobic core and a hydrophilic shell. Any hydrophobic or amphiphilic bioactive substances can be incorporated into these colloidal particles. The fact that the colloidal particles in microemulsions are much smaller than the wavelength of light means that they are typically optically clear or only slightly turbid. Because they are thermodynamically stable systems, microemulsions should form spontaneously when the different components are mixed together, but some mechanical mixing and/or heating may be required to facilitate this process. This is because there may be a kinetic energy barrier between the separated substances and the final microemulsion system that must be overcome.

Researchers have tried to optimize the formulation of curcumin-loaded microemulsions by varying the type and level of different substances used to fabricate them. For instance, one study reported that an optimum formulation consisted of DL-α-tocopherol (3.3 wt%), Tween 20 (53.8 wt%), and ethanol (6.6 wt%) [131]. This curcumin-loaded microemulsion had a clear yellow appearance, due to its small particle diameter (5 nm) and high curcumin level (14.6 mg/mL). These curcumin-loaded microemulsions remained stable when stored under refrigerator conditions (4 °C) for a month, with no significant change in particle size or curcumin concentration. This study also showed that the encapsulated curcumin had a relatively high permeability (around 70%) when tested using an in vitro method [131]. 

Another study showed that self-micro-emulsifying drug delivery systems (SMEDDSs) fabricated from oils and surfactants could be used to encapsulate and release curcumin [132]. In this case, the curcumin, oil, and surfactant are mixed together and then incorporated into capsules or pellets. This mixture spontaneously forms a microemulsion when it comes into contact with aqueous gastrointestinal fluids. These formulations exhibited excellent physical and chemical stability during storage, as well as leading to a 17-fold increase in the oral bioavailability of the curcumin (compared to the non-encapsulated form) using animal feeding studies [132]. Similar findings have been reported in other animal feeding studies using SMEDDSs [133]. Overall, these results suggest that microemulsions can be designed to encapsulate curcumin, improve its stability, and enhance its bioavailability. For food applications, however, the potential limitations of this approach are the high levels of synthetic surfactant required, which can cause problems with cost, taste, and toxicity. 

### 7.4. Nanoemulsions and Emulsions

Nanoemulsions and emulsions typically consist of two immiscible liquids stabilized by an emulsifier, and perhaps other ingredients, such as thickening agents, gelling agents, ripening inhibitors, or weighting agents [134]. Nanoemulsions typically contain droplets with a mean diameter between about 20 and 200 nm, whereas emulsions contain droplets with mean diameters greater than 200 nm [135]. As a result, nanoemulsions may appear clear to opaque, whereas emulsions nearly always appear opaque, due to differences in light scattering. Because nanoemulsions have a greater specific surface area (A_S_), they need more emulsifier to stabilize them, since A_S_ is proportional to the reciprocal of the mean particle diameter (d_32_). The physicochemical principles underlying the formation and stability of emulsions and nanoemulsions are the same, and so we will simply refer to them as “emulsions” in the remainder of this section. 

For encapsulation purposes, oil-in-water (O/W) emulsions are typically used because hydrophobic substances can be encapsulated inside the droplets and then introduced into aqueous-based food and beverage products. The small droplets found in emulsions can be produced using two different approaches: High- or low-intensity methods [135]. High-intensity methods employ mechanical devices that apply intense disruptive stresses to fluids, which cause large droplets to break down into smaller ones, and include high-shearing colloid mills, high-pressure homogenization, microfluidization, and sonication devices. Low-intensity methods rely on spontaneous droplet formation when oil–water–surfactant mixtures are exposed to particular conditions (compositions/temperatures), which includes phase inversion temperature and spontaneous emulsification methods [134,135].

Emulsion-based systems have been widely used for the encapsulation, stabilization, and delivery of curcumin. Curcumin-loaded emulsions have a yellow-orange clear to milky appearance, depending on the droplet concentration, droplet size, curcumin concentration, and solution pH [100]. As mentioned earlier, the emulsions look clear when the droplets are very small (< 40 nm) but milky when they are larger. The color of the emulsions depends on pH because of changes in the molecular conformation of the curcumin molecules discussed earlier [136]. 

Researchers have examined the impact of formulation parameters on the formation and functionality of curcumin-loaded emulsions, including the oil type, emulsifier type, and curcumin-solubilization method [137]. In particular, five oils (canola, corn, linseed, medium-chain triglycerides (MCTs), and sunflower, oil), four emulsifiers (lecithin, Tween 80, gum acacia, and whey protein), and three solubilization methods (heating, sonication, and microwaving) were investigated. Overall, heating was the most efficient method of dissolving curcumin in the oil phase, while MCT led to the highest curcumin content in the oil phase. The synthetic surfactant (Tween 80) produced curcumin-loaded MCT emulsions with relatively high curcumin contents, small particle diameters, high surface potentials, and good physical and chemical stabilities [137]. The natural emulsifiers could also produce curcumin-loaded emulsions, but they were not as stable as the ones produced by the synthetic surfactant. 

Another study compared three methods of producing curcumin-loaded emulsions from all-natural ingredients (corn oil and quillaja saponin): Conventional, heat-driven, and pH-driven methods. The conventional method involved dissolving powdered curcumin in an oil phase and then forming an emulsion by high-pressure homogenization. The heat-driven method involved preparing an emulsion first, and then heating it in the presence of powdered curcumin (100 C for 15 min). The pH-driven method involved mixing an acidified emulsion with an alkaline curcumin solution. The encapsulation efficiency was higher for the pH-driven method (93%) than the heat-driven method (76.2%) or conventional method (55.5%). The oil droplets in the curcumin-loaded emulsions produced using the pH-driven method had a high negative charge (−45 mV) and small particle diameter (180 nm). The bioaccessibility of the curcumin in the emulsions was determined using an in vitro model, and shown to be higher than non-encapsulated curcumin, as well as commercial curcumin supplements [55].

The physical and chemical stability of curcumin-loaded oil droplets has been shown to depend on their mean diameter (0.17, 0.52, or 14 µm) [138]. As expected, the rate of droplet creaming increased with increasing droplet size because of the increase in gravitational forces. Conversely, the chemical instability of the curcumin decreased with increasing droplet size, which was attributed to the reduction in the contact area between the oil and water phases. Overall, curcumin bioaccessibility did not depend strongly on the droplet size because of the competing effects of increased solubilization but decreased chemical stability of the curcumin in smaller droplets.

An animal (mouse) study examined the impact of the oil droplet size (50, 100, and 200 nm) on curcumin bioavailability, anti-inflammatory activity, and antiallergic effects [139]. Interestingly, a higher maximum plasma concentration and longer time to reach this value were found in the blood for the 100 nm droplets (C_max_ ≈ 11 ng/mL, T_max_ = 2 h) than for the 50 nm or 200 nm ones (C_max_ ≈ 5 ng/mL, T_max_ = 2 h). The 100 nm droplets also exhibited higher anti-inflammatory and antiallergic effects [139]. Again, these effects may be due to conflicting influences of the particle size on the bioaccessibility, stability, and absorption.

### 7.5. Solid Lipid Particles

Solid lipid nanoparticles (SLNs) are similar in composition and structure to oil-in-water emulsions, except that the lipid phase is crystalline at the application temperature [140,141]. The size of the lipid particles in the SLN suspensions vary from around 20 to 1000 nm depending on the formulation and preparation method used. Suspensions in the lower particle size range (< 50 nm) appear transparent, whereas those containing larger particles appear cloudy or opaque [142]. SLNs are typically prepared using the same methods as emulsions (high-pressure homogenization, sonication, or microfluidization), except homogenization is usually carried out at a temperature appreciably above the melting point of the lipid phase. In principle, a solidified lipid core is more effective at retaining and stabilizing curcumin than a liquified one. However, the system must be carefully designed to inhibit particle aggregation and curcumin expulsion when the lipid phase crystallizes [140,141]. In addition, solidified lipid phases are typically digested more slowly than liquid ones, which may lead to prolonged release of any encapsulated substances. 

Curcumin has been successfully encapsulated within SLNs coated by biopolymers (caseinate or caseinate/pectin) [143]. The size, charge, and stability of these SLNs could be controlled by optimizing the formulation and preparation methods used. Curcumin has also been encapsulated within SLNs coated by soy lecithin and Tween 80 [144]. Initially, the SLNs had a relatively small diameter (134 nm) and a high encapsulation efficiency for curcumin (92%). After incubation for 12 months at a refrigerated temperature (5 °C), the particle size only increased slightly (160 nm) and the curcumin content only decreased slightly (3%). These results suggest that the SLNs were suitable for curcumin encapsulation under low-temperature storage conditions. The researchers also investigated the pharmacokinetics of curcumin after oral administration to rats. Encapsulation of curcumin within the SLNs led to a 48-fold increase in the plasma concentration and a 39-fold increase in the area under the curve compared to a control (powdered curcumin in water), leading to an appreciable increase in oral bioavailability.

The efficacy of curcumin-loaded solid lipid nanoparticles (Cur-SLNs) and nanostructured lipid carriers (Cur-NLCs) has been compared using in vitro and in vivo studies [145]. Cur-SLNs were prepared using cetyl palmitate as a lipid source, leading to a highly regular crystalline structure, whereas Cur-NLCs were formulated using oleic acid and cholesterol as a lipid source, leading to a more irregular solid structure. The storage stability, antioxidant activity, pharmacokinetics, and cytotoxicity of the Cur-SLNs and Cur-NLCs were then compared. The entrapment efficiency and storage stability of the Cur-NLCs were higher than that of the Cur-SLNs, but their antioxidant activities were similar. In an animal (rat) pharmacokinetic study, the area under the curve (AUC) in the plasma was 5-fold and 2-fold higher for Cur-NLCs and Cur-SLNs than the control, indicating that both delivery systems increased the bioavailability. Thus, NLCs appeared to be more effective at increasing the efficacy of curcumin than SLNs. In vivo studies with humans have shown that curcumin-loaded SLNs can appreciably increase the bioavailability of curcumin [146].

### 7.6. Biopolymer Particles

Biopolymer particles are typically assembled from proteins and/or polysaccharides using an appropriate method. The method employed depends on the nature of the biopolymers themselves, as well as the required characteristics of the colloidal particles formed (such as the composition, size, shape, charge, and stability). The final particles usually contain biopolymer molecules held together by attractive forces, such as hydrophobic, electrostatic, or hydrogen bonding interactions. The most common methods of inducing the assembly of biopolymer molecules are ionotropic, cold-set, heat-set, or enzymatic gelation. The most common particle-forming methods include injection, emulsion templating, electrostatic complexation, antisolvent precipitation, and thermodynamic incompatibility methods [147]. Biopolymer particles can be prepared with mean diameters ranging from around 100 nm to 1 mm depending on the fabrication method used.

Biopolymer particles can be formed from proteins or polysaccharides that have antioxidant properties, thereby protecting labile nutraceuticals from chemical degradation [98]. They can also be designed to retain nutraceuticals under one set of environmental conditions but then release them under another set [147]. For example, biopolymer particles could be designed to retain curcumin during storage, mouth, and stomach conditions but then release it in under small intestinal conditions. 

Curcumin-loaded lipid droplets have been encapsulated within hydrogel beads made from either alginate (370 µm) or chitosan (255 µm) using an injection-gelation method [148]. The anionic alginate was cross-linked with cationic calcium ions, whereas the cationic chitosan was cross-linked with anionic tripolyphosphate ions. Interestingly, encapsulating the curcumin-loaded lipid droplets within the hydrogel beads actually reduced its chemical stability. Moreover, there was some swelling and shape changes in the beads during storage. Curcumin has also been encapsulated within wheat protein microgels (510 nm) formed by heating the system at a controlled pH [149]. The protein microgels had a relatively high encapsulation efficiency (90%), good sedimentation stability, and high antioxidant activity, which may be important for commercial applications.

Encapsulating curcumin within biopolymer microgels may be an advantage in applications where sustained or triggered release is required. An in vitro study showed a faster and higher release of curcumin from free oil droplets than from oil droplets trapped in either carrageenan beads or alginate beads [150]. Similarly, the release of curcumin under simulated gastrointestinal conditions has been shown to be reduced when it is encapsulated within whey protein microgels [149]. These results suggest that biopolymer microgels may be useful for prolonging the release of curcumin, rather than increasing its bioavailability.

The water dispersibility and antioxidant activity of curcumin have been increased by incorporating it within the hydrophobic cores of casein micelles [151]. In a human study, it was shown that the oral bioavailability of curcumin could be increased substantially by loading it into γ-cyclodextrin complexes [152].

### 7.7. Nature-Derived Colloidal Particles

The growing interest in developing more sustainable and healthy food products has led many food scientists to explore the possibility of using nature-derived colloidal particles to encapsulate curcumin. Recently, it was shown that curcumin could be encapsulated within the milk fat globules in bovine milk [136] and the oil bodies in plant-based milks [152]. The curcumin was loaded into these nature-derived colloidal particles using the pH-shift method. First, curcumin is dissolved in a highly alkaline aqueous solution (pH 12), which is then mixed with an acidified milk product. The final pH of the mixed system is designed to be neutral or below, leading to a decrease in the water solubility of the curcumin, which causes it to move into the hydrophobic core of the colloidal particles (milk fat globules or oil bodies). The authors showed that the curcumin-loaded milks had good storage stability and a high curcumin bioaccessibility, as determined by an in vitro digestion method [136,152].

## 8. Conclusions

Curcumin has been used as an edible health-promoting substance for thousands of years as part of traditional medicinal practices in Asia. More recently, modern scientific methods have demonstrated that curcumin exhibits a broad spectrum of biological activities that may be beneficial to human health, including antioxidant, antimicrobial, anti-inflammatory, and antitumor activities. Even so, there are a number of challenges that have to be addressed when formulating curcumin-based functional foods or therapeutics, including its low water solubility, chemical stability, and bioavailability. In this article, we highlighted some of the methods that can be used to overcome these problems, including antioxidant, encapsulation, and storage strategies. In particular, we focused on the utilization of colloidal delivery systems, such as micelles, liposomes, microemulsions, emulsions, solid lipid nanoparticles, biopolymer particles, and nature-derived colloidal particles. Each of these delivery systems has its own advantages and disadvantages for specific applications and it is important to select the most appropriate formulation. For instance, there are differences in the appearances, textures, mouthfeels, flavors, shelf-lives, and environmental histories of different curcumin-fortified functional food products (such as soft drinks, milky drinks, sauces, dressings, and bakery goods), which require different kinds of encapsulation technologies. In the future, it will be important to compare different formulations in terms of their cost, ease of manufacture, robustness, pharmacokinetics, bioavailability, bioactivity, sustainability, and environmental impact. The most suitable formulation for a specific application can then be selected.

## Abbreviations

C4-2B	C4-2 Bone metastatic
*E. coli*	Escherichia coli
*E. faecalis*	Enterococcus faecalis
HCT 116	Human Colorectal Carcinoma cell lines
IL	Interleukin
LNCaP	Lymph Node Carcinoma of the Prostate
NFkB	Nuclear Factor Kappa B
P. aeruginosa	Pseudomonas aeruginosa
Rko	Rectal carcinoma cell line
*S. autrus*	Staphylococcus aureus
TNF-a	Tumor Necrosis Factor Alpha

## References

[1] Sharma R., Gescher A., Steward W. (2005). Curcumin: The story so far. Eur. J. Cancer.

[2] Shahidi F., Naczk M. (2003). Phenolics in Food and Nutraceuticals.

[3] Heger M., van Golen R.F., Broekgaarden M., Michel M.C. (2014). The molecular basis for the pharmacokinetics and pharmacodynamics of curcumin and its metabolites in relation to cancer. Pharmacol. Rev..

[4] Priyadarsini K.I. (2014). The chemistry of curcumin: From extraction to therapeutic agent. Molecules.

[5] Jurenka J.S. (2009). Anti-inflammatory properties of curcumin, a major constituent of curcuma longa: A review of preclinical and clinical research. Altern. Med. Rev..

[6] Menon V.P., Sudheer A.R. (2007). Antioxidant and anti-inflammatory properties of curcumin. The Molecular Targets and Therapeutic Uses of Curcumin in Health and Disease.

[7] Ak T., Gülçin İ. (2008). Antioxidant and radical scavenging properties of curcumin. Chem. Biol. Interact..

[8] Zorofchian Moghadamtousi S., Abdul Kadir H., Hassandarvish P., Tajik H., Abubakar S., Zandi K. (2014). A review on antibacterial, antiviral, and antifungal activity of curcumin. BioMed Res. Int..

[9] Martins C., Da Silva D., Neres A., Magalhaes T., Watanabe G., Modolo L., Sabino A., De Fátima A., De Resende M. (2008). Curcumin as a promising antifungal of clinical interest. J. Antimicrob. Chemother..

[10] Bar-Sela G., Epelbaum R., Schaffer M. (2010). Curcumin as an anti-cancer agent: Review of the gap between basic and clinical applications. Curr. Med. Chem..

[11] Naksuriya O., Okonogi S., Schiffelers R.M., Hennink W.E. (2014). Curcumin nanoformulations: A review of pharmaceutical properties and preclinical studies and clinical data related to cancer treatment. Biomaterials.

[12] Anand P., Kunnumakkara A.B., Newman R.A., Aggarwal B.B. (2007). Bioavailability of curcumin: Problems and promises. Mol. Pharm..

[13] Tønnesen H.H., Másson M., Loftsson T. (2002). Studies of curcumin and curcuminoids. Xxvii. Cyclodextrin complexation: Solubility, chemical and photochemical stability. Int. J. Pharm..

[14] Kharat M., Du Z., Zhang G., McClements D.J. (2017). Physical and chemical stability of curcumin in aqueous solutions and emulsions: Impact of ph, temperature, and molecular environment. J. Agric. Food Chem..

[15] McClements D.J., Decker E.A., Park Y., Weiss J. (2009). Structural design principles for delivery of bioactive components in nutraceuticals and functional foods. Crit. Rev. Food Sci. Nutr..

[16] Garti N. (2008). Delivery and Controlled Release of Bioactives in Foods and Nutraceuticals.

[17] Zhang Z., Zhang R., Decker E.A., McClements D.J. (2015). Development of food-grade filled hydrogels for oral delivery of lipophilic active ingredients: Ph-triggered release. Food Hydrocoll..

[18] McClements D., Decker E., Weiss J. (2007). Emulsion-based delivery systems for lipophilic bioactive components. J. Food Sci..

[19] Lee W.-H., Loo C.-Y., Bebawy M., Luk F., Mason R.S., Rohanizadeh R. (2013). Curcumin and its derivatives: Their application in neuropharmacology and neuroscience in the 21st century. Curr. Neuropharmacol..

[20] Bhatia N.K., Kishor S., Katyal N., Gogoi P., Narang P., Deep S. (2016). Effect of ph and temperature on conformational equilibria and aggregation behaviour of curcumin in aqueous binary mixtures of ethanol. RSC Adv..

[21] Manolova Y., Deneva V., Antonov L., Drakalska E., Momekova D., Lambov N. (2014). The effect of the water on the curcumin tautomerism: A quantitative approach. Spectrochim. Acta Part A Mol. Biomol. Spectrosc..

[22] Murugan P., Pari L. (2007). Influence of tetrahydrocurcumin on hepatic and renal functional markers and protein levels in experimental type 2 diabetic rats. Basic Clin. Pharmacol. Toxicol..

[23] Willcox J.K., Ash S.L., Catignani G.L. (2004). Antioxidants and prevention of chronic disease. Crit. Rev. Food Sci. Nutr..

[24] Barclay L.R.C., Vinqvist M.R., Mukai K., Goto H., Hashimoto Y., Tokunaga A., Uno H. (2000). On the antioxidant mechanism of curcumin: Classical methods are needed to determine antioxidant mechanism and activity. Org. Lett..

[25] Jayaprakasha G., Rao L.J., Sakariah K. (2006). Antioxidant activities of curcumin, demethoxycurcumin and bisdemethoxycurcumin. Food Chem..

[26] Goel A., Kunnumakkara A.B., Aggarwal B.B. (2008). Curcumin as “curecumin”: From kitchen to clinic. Biochem. Pharmacol..

[27] Arun N., Nalini N. (2002). Efficacy of turmeric on blood sugar and polyol pathway in diabetic albino rats. Plant Foods Hum. Nutr..

[28] Chandran B., Goel A. (2012). A randomized, pilot study to assess the efficacy and safety of curcumin in patients with active rheumatoid arthritis. Phytother. Res..

[29] Anna K.T., Suhana M., Das S., Faizah O., Hamzaini A. (2011). Anti-inflammatory effect of curcuma longa (turmeric) on collagen-induced arthritis: An anatomico-radiological study. Clin. Ter..

[30] Yang Q.Q., Farha A.K., Kim G., Gul K., Gan R.Y., Corke H. (2020). Antimicrobial and anticancer applications and related mechanisms of curcumin-mediated photodynamic treatments. Trends Food Sci. Technol..

[31] Gupta S.C., Sung B., Kim J.H., Prasad S., Li S.Y., Aggarwal B.B. (2013). Multitargeting by turmeric, the golden spice: From kitchen to clinic. Mol. Nutr. Food Res..

[32] Vaughn A.R., Haas K.N., Burney W., Andersen E., Clark A.K., Crawford R., Sivamani R.K. (2017). Potential role of curcumin against biofilm-producing organisms on the skin: A review. Phytother. Res..

[33] Tyagi P., Singh M., Kumari H., Kumari A., Mukhopadhyay K. (2015). Bactericidal activity of curcumin i is associated with damaging of bacterial membrane. PLoS ONE.

[34] Tomeh M.A., Hadianamrei R., Zhao X. (2019). A review of curcumin and its derivatives as anticancer agents. Int. J. Mol. Sci..

[35] Arbiser J.L., Klauber N., Rohan R., van Leeuwen R., Huang M.-T., Fisher C., Flynn E., Byers H.R. (1998). Curcumin is an in vivo inhibitor of angiogenesis. Mol. Med..

[36] Teiten M.-H., Gaascht F., Eifes S., Dicato M., Diederich M. (2010). Chemopreventive potential of curcumin in prostate cancer. Genes Nutr..

[37] Dorai T., Dutcher J.P., Dempster D.W., Wiernik P.H. (2004). Therapeutic potential of curcumin in prostate cancer—IV: Interference with the osteomimetic properties of hormone refractory c4-2b prostate cancer cells. Prostate.

[38] Liu Q., Loo W.T., Sze S., Tong Y. (2009). Curcumin inhibits cell proliferation of mda-mb-231 and bt-483 breast cancer cells mediated by down-regulation of nfκb, cyclind and mmp-1 transcription. Phytomedicine.

[39] Mudduluru G., George-William J.N., Muppala S., Asangani I.A., Kumarswamy R., Nelson L.D., Allgayer H. (2011). Curcumin regulates mir-21 expression and inhibits invasion and metastasis in colorectal cancer. Biosci. Rep..

[40] Kunnumakkara A.B., Bordoloi D., Harsha C., Banik K., Gupta S.C., Aggarwal B.B. (2017). Curcumin mediates anticancer effects by modulating multiple cell signaling pathways. Clin. Sci..

[41] Zhou H.Y., Beevers C.S., Huang S.L. (2011). The targets of curcumin. Curr. Drug Targets.

[42] Cheng A.-L., Hsu C.-H., Lin J.-K., Hsu M.-M., Ho Y.-F., Shen T.-S., Ko J.-Y., Lin J.-T., Lin B.-R., Ming-Shiang W. (2001). Phase i clinical trial of curcumin, a chemopreventive agent, in patients with high-risk or pre-malignant lesions. Anticancer Res..

[43] Lao C.D., Ruffin M.T., Normolle D., Heath D.D., Murray S.I., Bailey J.M., Boggs M.E., Crowell J., Rock C.L., Brenner D.E. (2006). Dose escalation of a curcuminoid formulation. BMC Complementary Altern. Med..

[44] Rodriguez J.C., Santibanez D., Narayanan S., Dave A. (2008). Ginger and curcumin in cancer prevention and health promotion. Bot. Med. Clin. Pract..

[45] Authority E.F.S. (2014). Refined exposure assessment for curcumin (e 100). EFSA J..

[46] Hewlings S., Kalman D. (2017). Curcumin: A review of its’ effects on human health. Foods.

[47] DiSilvestro R.A., Joseph E., Zhao S., Bomser J. (2012). Diverse effects of a low dose supplement of lipidated curcumin in healthy middle aged people. Nutr. J..

[48] Cianfruglia L., Minnelli C., Laudadio E., Scire A., Armeni T. (2019). Side effects of curcumin: Epigenetic and antiproliferative implications for normal dermal fibroblast and breast cancer cells. Antioxidants.

[49] Araiza-Calahorra A., Akhtar M., Sarkar A. (2018). Recent advances in emulsion-based delivery approaches for curcumin: From encapsulation to bioaccessibility. Trends Food Sci. Technol..

[50] Grynkiewicz G., Ślifirski P. (2012). Curcumin and curcuminoids in quest for medicinal status. Acta Biochim. Pol..

[51] Bernabé-Pineda M., Ramĺrez-Silva M.a.T., Romero-Romo M., González-Vergara E., Rojas-Hernández A. (2004). Determination of acidity constants of curcumin in aqueous solution and apparent rate constant of its decomposition. Spectrochim. Acta Part A Mol. Biomol. Spectrosc..

[52] Schneider C., Gordon O.N., Edwards R.L., Luis P.B. (2015). Degradation of curcumin: From mechanism to biological implications. J. Agric. Food Chem..

[53] Wang Y.-J., Pan M.-H., Cheng A.-L., Lin L.-I., Ho Y.-S., Hsieh C.-Y., Lin J.-K. (1997). Stability of curcumin in buffer solutions and characterization of its degradation products. J. Pharm. Biomed. Anal..

[54] Zheng B., Peng S., Zhang X., McClements D.J. (2018). Impact of delivery system type on curcumin bioaccessibility: Comparison of curcumin-loaded nanoemulsions with commercial curcumin supplements. J. Agric. Food Chem..

[55] Nelson K.M., Dahlin J.L., Bisson J., Graham J., Pauli G.F., Walters M.A. (2017). The essential medicinal chemistry of curcumin: Miniperspective. J. Med. Chem..

[56] Priyadarsini K.I. (2009). Photophysics, photochemistry and photobiology of curcumin: Studies from organic solutions, bio-mimetics and living cells. J. Photochem. Photobiol. C Photochem. Rev..

[57] Wright L., Frye J.B., Gorti B., Timmermann B.N., Funk J.L. (2013). Bioactivity of turmeric-derived curcuminoids and related metabolites in breast cancer. Curr. Pharm. Des..

[58] Ogiwara T., Satoh K., Kadoma Y., Murakami Y., Unten S., Atsumi T., Sakagami H., Fujisawa S. (2002). Radical scavenging activity and cytotoxicity of ferulic acid. Anticancer Res..

[59] Tai A., Sawano T., Yazama F., Ito H. (2011). Evaluation of antioxidant activity of vanillin by using multiple antioxidant assays. Biochim. Biophys. Acta.

[60] Gordon O.N., Schneider C. (2012). Vanillin and ferulic acid: Not the major degradation products of curcumin. Trends Mol. Med..

[61] Gordon O.N., Luis P.B., Sintim H.O., Schneider C. (2015). Unraveling curcumin degradation autoxidation proceeds through spiroepoxide and vinylether intermediates en route to the main bicyclopentadione. J. Biol. Chem..

[62] Griesser M., Pistis V., Suzuki T., Tejera N., Pratt D.A., Schneider C. (2011). Autoxidative and cyclooxygenase-2 catalyzed transformation of the dietary chemopreventive agent curcumin. J. Biol. Chem..

[63] Sanidad K.Z., Zhu J., Wang W., Du Z., Zhang G. (2016). Effects of stable degradation products of curcumin on cancer cell proliferation and inflammation. J. Agric. Food Chem..

[64] McClements D.J., Li F., Xiao H. (2015). The nutraceutical bioavailability classification scheme: Classifying nutraceuticals according to factors limiting their oral bioavailability. Annu. Rev. Food Sci. Technol..

[65] Ravindranath V., Chandrasekhara N. (1980). Absorption and tissue distribution of curcumin in rats. Toxicology.

[66] Sanidad K.Z., Sukamtoh E., Xiao H., McClements D.J., Zhang G.D. (2019). Curcumin: Recent advances in the development of strategies to improve oral bioavailability. Annu. Rev. Food Sci. Technol..

[67] Jain G., Patil U.K. (2015). Strategies for enhancement of bioavailability of medicinal agents with natural products. Int. J. Pharm. Sci. Res..

[68] Mollazadeh S., Sahebkar A., Hadizadeh F., Behravan J., Arabzadeh S. (2018). Structural and functional aspects of p-glycoprotein and its inhibitors. Life Sci..

[69] Zhou S.F., Lim L.Y., Chowbay B. (2004). Herbal modulation of p-glycoprotein. Drug Metab. Rev..

[70] Singh D.V., Godbole M.M., Misra K. (2013). A plausible explanation for enhanced bioavailability of p-gp substrates in presence of piperine: Simulation for next generation of p-gp inhibitors. J. Mol. Modeling.

[71] Prasad S., Tyagi A.K., Aggarwal B.B. (2014). Recent developments in delivery, bioavailability, absorption and metabolism of curcumin: The golden pigment from golden spice. Cancer Res. Treat..

[72] Ireson C.R., Jones D.J., Orr S., Coughtrie M.W., Boocock D.J., Williams M.L., Farmer P.B., Steward W.P., Gescher A.J. (2002). Metabolism of the cancer chemopreventive agent curcumin in human and rat intestine. Cancer Epidemiol. Prev. Biomark..

[73] Ireson C., Orr S., Jones D.J., Verschoyle R., Lim C.-K., Luo J.-L., Howells L., Plummer S., Jukes R., Williams M. (2001). Characterization of metabolites of the chemopreventive agent curcumin in human and rat hepatocytes and in the rat in vivo, and evaluation of their ability to inhibit phorbol ester-induced prostaglandin e2 production. Cancer Res..

[74] Asai A., Miyazawa T. (2000). Occurrence of orally administered curcuminoid as glucuronide and glucuronide/sulfate conjugates in rat plasma. Life Sci..

[75] Sharma R.A., Euden S.A., Platton S.L., Cooke D.N., Shafayat A., Hewitt H.R., Marczylo T.H., Morgan B., Hemingway D., Plummer S.M. (2004). Phase i clinical trial of oral curcumin: Biomarkers of systemic activity and compliance. Clin. Cancer Res..

[76] Dubey S.K., Sharma A.K., Narain U., Misra K., Pati U. (2008). Design, synthesis and characterization of some bioactive conjugates of curcumin with glycine, glutamic acid, valine and demethylenated piperic acid and study of their antimicrobial and antiproliferative properties. Eur. J. Med. Chem..

[77] Huang Y., Cao S., Zhang Q., Zhang H., Fan Y., Qiu F., Kang N. (2018). Biological and pharmacological effects of hexahydrocurcumin, a metabolite of curcumin. Arch. Biochem. Biophys..

[78] Srimuangwong K., Tocharus C., Chintana P.Y., Suksamrarn A., Tocharus J. (2012). Hexahydrocurcumin enhances inhibitory effect of 5-fluorouracil on ht-29 human colon cancer cells. World J. Gastroenterol..

[79] Chen C.-Y., Yang W.-L., Kuo S.-Y. (2011). Cytotoxic activity and cell cycle analysis of hexahydrocurcumin on sw 480 human colorectal cancer cells. Nat. Prod. Commun..

[80] Zhang Z., Luo D., Xie J., Lin G., Zhou J., Liu W., Li H., Yi T., Su Z., Chen J. (2018). Octahydrocurcumin, a final hydrogenated metabolite of curcumin, possesses superior anti-tumor activity through induction of cellular apoptosis. Food Funct..

[81] Luo D.-D., Chen J.-F., Liu J.-J., Xie J.-H., Zhang Z.-B., Gu J.-Y., Zhuo J.-Y., Huang S., Su Z.-R., Sun Z.-H. (2019). Tetrahydrocurcumin and octahydrocurcumin, the primary and final hydrogenated metabolites of curcumin, possess superior hepatic-protective effect against acetaminophen-induced liver injury: Role of cyp2e1 and keap1-nrf2 pathway. Food Chem. Toxicol..

[82] Shoji M., Nakagawa K., Watanabe A., Tsuduki T., Yamada T., Kuwahara S., Kimura F., Miyazawa T. (2014). Comparison of the effects of curcumin and curcumin glucuronide in human hepatocellular carcinoma hepg2 cells. Food Chem..

[83] Shen L., Liu C.-C., An C.-Y., Ji H.-F. (2016). How does curcumin work with poor bioavailability? Clues from experimental and theoretical studies. Sci. Rep..

[84] Perkins S., Verschoyle R.D., Hill K., Parveen I., Threadgill M.D., Sharma R.A., Williams M.L., Steward W.P., Gescher A.J. (2002). Chemopreventive efficacy and pharmacokinetics of curcumin in the min/+ mouse, a model of familial adenomatous polyposis. Cancer Epidemiol. Prev. Biomark..

[85] Suresh D., Srinivasan K. (2010). Tissue distribution & elimination of capsaicin, piperine & curcumin following oral intake in rats. Indian J. Med. Res..

[86] Ravindranath V., Chandrasekhara N. (1981). Metabolism of curcumn-studies with [3 h] curcumin. Toxicology.

[87] Pan M.-H., Huang T.-M., Lin J.-K. (1999). Biotransformation of curcumin through reduction and glucuronidation in mice. Drug Metab. Dispos..

[88] Kakran M., Sahoo N.G., Tan I.-L., Li L. (2012). Preparation of nanoparticles of poorly water-soluble antioxidant curcumin by antisolvent precipitation methods. J. Nanoparticle Res..

[89] Yadav D., Kumar N. (2014). Nanonization of curcumin by antisolvent precipitation: Process development, characterization, freeze drying and stability performance. Int. J. Pharm..

[90] Patel A., Hu Y., Tiwari J.K., Velikov K.P. (2010). Synthesis and characterisation of zein-curcumin colloidal particles. Soft Mater.

[91] Khan F.I., Ghoshal A.K. (2000). Removal of volatile organic compounds from polluted air. J. Loss Prev. Process Ind..

[92] Mozafari M.R. (2005). Liposomes: An overview of manufacturing techniques. Cell. Mol. Biol. Lett..

[93] Lesoin L., Crampon C., Boutin O., Badens E. (2011). Preparation of liposomes using the supercritical anti-solvent (sas) process and comparison with a conventional method. J. Supercrit. Fluids.

[94] Ginty P.J., Whitaker M.J., Shakesheff K.M., Howdle S.M. (2005). Drug delivery goes supercritical. Mater. Today.

[95] Peng S., Li Z., Zou L., Liu W., Liu C., McClements D.J. (2018). Enhancement of curcumin bioavailability by encapsulation in sophorolipid-coated nanoparticles: An in vitro and in vivo study. J. Agric. Food Chem..

[96] Cheng C., Peng S., Li Z., Zou L., Liu W., Liu C. (2017). Improved bioavailability of curcumin in liposomes prepared using a ph-driven, organic solvent-free, easily scalable process. RSC Adv..

[97] Pan K., Luo Y., Gan Y., Baek S.J., Zhong Q. (2014). Ph-driven encapsulation of curcumin in self-assembled casein nanoparticles for enhanced dispersibility and bioactivity. Soft Matter.

[98] Zhou M., Wang T., Hu Q., Luo Y. (2016). Low density lipoprotein/pectin complex nanogels as potential oral delivery vehicles for curcumin. Food Hydrocoll..

[99] Zheng B., Zhang X., Peng S., McClements D.J. (2019). Impact of delivery system format on curcumin bioaccessibility: Nanocrystals, nanoemulsion droplets, and natural oil bodies. Food Funct..

[100] Cabrera-Trujillo M.A., Sotelo-Díaz L.I., Quintanilla-Carvajal M.X. (2016). Effect of amplitude and pulse in low frequency ultrasound on oil/water emulsions. DYNA.

[101] Kim H.N., Suslick K.S. (2018). The effects of ultrasound on crystals: Sonocrystallization and sonofragmentation. Crystals.

[102] Zou L., Zheng B., Zhang R., Zhang Z., Liu W., Liu C., Xiao H., McClements D.J. (2016). Food matrix effects on nutraceutical bioavailability: Impact of protein on curcumin bioaccessibility and transformation in nanoemulsion delivery systems and excipient nanoemulsions. Food Biophys..

[103] Zou L., Zheng B., Zhang R., Zhang Z., Liu W., Liu C., Zhang G., Xiao H., McClements D.J. (2016). Influence of lipid phase composition of excipient emulsions on curcumin solubility, stability, and bioaccessibility. Food Biophys..

[104] Zhu J.L., Sanidad K.Z., Sukamtoh E., Zhang G.D. (2017). Potential roles of chemical degradation in the biological activities of curcumin. Food Funct..

[105] Kharat M., Skrzynski M., Decker E.A., McClements D.J. (2020). Enhancement of chemical stability of curcumin-enriched oil-in-water emulsions: Impact of antioxidant type and concentration. Food Chem..

[106] Zou L.Q., Zheng B.J., Zhang R.J., Zhang Z.P., Liu W., Liu C.M., Xiao H., McClements D.J. (2016). Food-grade nanoparticles for encapsulation, protection and delivery of curcumin: Comparison of lipid, protein, and phospholipid nanoparticles under simulated gastrointestinal conditions. RSC Adv..

[107] Dai L., Zhou H.L., Wei Y., Gao Y.X., McClements D.J. (2019). Curcumin encapsulation in zein-rhamnolipid composite nanoparticles using a ph-driven method. Food Hydrocoll..

[108] Yallapu M.M., Jaggi M., Chauhan S.C. (2012). Curcumin nanoformulations: A future nanomedicine for cancer. Drug Discov. Today..

[109] del Castillo M.L.R., Lopez-Tobar E., Sanchez-Cortes S., Flores G., Blanch G.P. (2015). Stabilization of curcumin against photodegradation by encapsulation in gamma-cyclodextrin: A study based on chromatographic and spectroscopic (raman and uv-visible) data. Vib. Spectrosc..

[110] Price L.C., Buescher R.W. (1996). Decomposition of turmeric curcuminoids as affected by light, solvent and oxygen. J. Food Biochem..

[111] Higaki K., Yata T., Sone M., Ogawara K., Kimura T. (2001). Estimation of absorption enhancement by medium-chain fatty acids in rat large intestine. Res. Commun. Mol. Pathol. Pharmacol..

[112] Aungst B.J. (2000). Intestinal permeation enhancers. J. Pharm. Sci..

[113] Patra A.K., Amasheh S., Aschenbach J.R. (2019). Modulation of gastrointestinal barrier and nutrient transport function in farm animals by natural plant bioactive compounds—A comprehensive review. Crit. Rev. Food Sci. Nutr..

[114] McClements D.J. (2014). Nanoparticle- and Microparticle-Based Delivery Systems: Encapsulation, Protection and Release of Active Components.

[115] McClements D.J. (2015). Enhancing nutraceutical bioavailability through food matrix design. Curr. Opin. Food Sci..

[116] Dordevic V., Balanc B., Belscak-Cvitanovic A., Levic S., Trifkovic K., Kalusevic A., Kostic I., Komes D., Bugarski B., Nedovic V. (2015). Trends in encapsulation technologies for delivery of food bioactive compounds. Food Eng. Rev..

[117] Wang Z.L. (2011). Bioavailability of organic compounds solubilized in nonionic surfactant micelles. Appl. Microbiol. Biotechnol..

[118] Kimpel F., Schmitt J.J. (2015). Review: Milk proteins as nanocarrier systems for hydrophobic nutraceuticals. J. Food Sci..

[119] Livney Y.D. (2010). Milk proteins as vehicles for bioactives. Curr. Opin. Colloid Interface Sci..

[120] Richtering W. (2001). Rheology and shear induced structures in surfactant solutions. Curr. Opin. Colloid Interface Sci..

[121] Torchilin V.P. (2007). Micellar nanocarriers: Pharmaceutical perspectives. Pharm. Res..

[122] Wang X.Y., Gao Y. (2018). Effects of length and unsaturation of the alkyl chain on the hydrophobic binding of curcumin with tween micelles. Food Chem..

[123] Pan K., Zhong Q., Baek S.J. (2013). Enhanced dispersibility and bioactivity of curcumin by encapsulation in casein nanocapsules. J. Agric. Food Chem..

[124] Schiborr C., Kocher A., Behnam D., Jandasek J., Toelstede S., Frank J. (2014). The oral bioavailability of curcumin from micronized powder and liquid micelles is significantly increased in healthy humans and differs between sexes. Mol. Nutr. Food Res..

[125] Akbarzadeh A., Rezaei-Sadabady R., Davaran S., Joo S.W., Zarghami N., Hanifehpour Y., Samiei M., Kouhi M., Nejati-Koshki K. (2013). Liposome: Classification, preparation, and applications. Nanoscale Res. Lett..

[126] Chen X., Zou L.-Q., Niu J., Liu W., Peng S.-F., Liu C.-M. (2015). The stability, sustained release and cellular antioxidant activity of curcumin nanoliposomes. Molecules.

[127] Jin H.-H., Lu Q., Jiang J.-G. (2016). Curcumin liposomes prepared with milk fat globule membrane phospholipids and soybean lecithin. J. Dairy Sci..

[128] Takahashi M., Uechi S., Takara K., Asikin Y., Wada K. (2009). Evaluation of an oral carrier system in rats: Bioavailability and antioxidant properties of liposome-encapsulated curcumin. J. Agric. Food Chem..

[129] Li C., Zhang Y., Su T., Feng L., Long Y., Chen Z. (2012). Silica-coated flexible liposomes as a nanohybrid delivery system for enhanced oral bioavailability of curcumin. Int. J. Nanomed..

[130] Bergonzi M., Hamdouch R., Mazzacuva F., Isacchi B., Bilia A. (2014). Optimization, characterization and in vitro evaluation of curcumin microemulsions. LWT Food Sci. Technol..

[131] Setthacheewakul S., Mahattanadul S., Phadoongsombut N., Pichayakorn W., Wiwattanapatapee R. (2010). Development and evaluation of self-microemulsifying liquid and pellet formulations of curcumin, and absorption studies in rats. Eur. J. Pharm. Biopharm..

[132] Hu L., Jia Y., Niu F., Jia Z., Yang X., Jiao K. (2012). Preparation and enhancement of oral bioavailability of curcumin using microemulsions vehicle. J. Agric. Food Chem..

[133] McClements D.J. (2015). Food Emulsions: Principles, Practices, and Techniques.

[134] McClements D.J. (2012). Nanoemulsions versus microemulsions: Terminology, differences, and similarities. Soft Matter.

[135] Zheng B., Lin H., Zhang X., McClements D.J. (2019). Fabrication of curcumin-loaded dairy milks using the ph-shift method: Formation, stability, and bioaccessibility. J. Agric. Food Chem..

[136] Ma P., Zeng Q., Tai K., He X., Yao Y., Hong X., Yuan F. (2017). Preparation of curcumin-loaded emulsion using high pressure homogenization: Impact of oil phase and concentration on physicochemical stability. LWT.

[137] Zou L., Zheng B., Liu W., Liu C., Xiao H., McClements D.J. (2015). Enhancing nutraceutical bioavailability using excipient emulsions: Influence of lipid droplet size on solubility and bioaccessibility of powdered curcumin. J. Funct. Foods.

[138] Onodera T., Kuriyama I., Andoh T., Ichikawa H., Sakamoto Y., Lee-Hiraiwa E., Mizushina Y. (2015). Influence of particle size on the in vitro and in vivo anti-inflammatory and anti-allergic activities of a curcumin lipid nanoemulsion. Int. J. Mol. Med..

[139] Mishra V., Bansal K.K., Verma A., Yadav N., Thakur S., Sudhakar K., Rosenholm J.M. (2018). Solid lipid nanoparticles: Emerging colloidal nano drug delivery systems. Pharmaceutics.

[140] Müller R.H., Radtke M., Wissing S.A. (2002). Solid lipid nanoparticles (sln) and nanostructured lipid carriers (nlc) in cosmetic and dermatological preparations. Adv. Drug Deliv. Rev..

[141] Helgason T., Salminen H., Kristbergsson K., McClements D.J., Weiss J. (2015). Formation of transparent solid lipid nanoparticles by microfluidization: Influence of lipid physical state on appearance. J. Colloid Interface Sci..

[142] Xue J., Wang T., Hu Q., Zhou M., Luo Y. (2018). Insight into natural biopolymer-emulsified solid lipid nanoparticles for encapsulation of curcumin: Effect of loading methods. Food Hydrocoll..

[143] Kakkar V., Singh S., Singla D., Kaur I.P. (2011). Exploring solid lipid nanoparticles to enhance the oral bioavailability of curcumin. Mol. Nutr. Food Res..

[144] Sadegh Malvajerd S., Azadi A., Izadi Z., Kurd M., Dara T., Dibaei M., Sharif Zadeh M., Akbari Javar H., Hamidi M. (2018). Brain delivery of curcumin using solid lipid nanoparticles and nanostructured lipid carriers: Preparation, optimization, and pharmacokinetic evaluation. ACS Chem. Neurosci..

[145] Gota V.S., Maru G.B., Soni T.G., Gandhi T.R., Kochar N., Agarwal M.G. (2010). Safety and pharmacokinetics of a solid lipid curcumin particle formulation in osteosarcoma patients and healthy volunteers. J. Agric. Food Chem..

[146] McClements D.J. (2017). Recent progress in hydrogel delivery systems for improving nutraceutical bioavailability. Food Hydrocoll..

[147] Zheng B., Zhang Z., Chen F., Luo X., McClements D.J. (2017). Impact of delivery system type on curcumin stability: Comparison of curcumin degradation in aqueous solutions, emulsions, and hydrogel beads. Food Hydrocoll..

[148] Mohammadian M., Salami M., Momen S., Alavi F., Emam-Djomeh Z. (2019). Fabrication of curcumin-loaded whey protein microgels: Structural properties, antioxidant activity, and in vitro release behavior. LWT.

[149] Zhang Z., Zhang R., Zou L., Chen L., Ahmed Y., Al Bishri W., Balamash K., McClements D.J. (2016). Encapsulation of curcumin in polysaccharide-based hydrogel beads: Impact of bead type on lipid digestion and curcumin bioaccessibility. Food Hydrocoll..

[150] Esmaili M., Ghaffari S.M., Moosavi-Movahedi Z., Atri M.S., Sharifizadeh A., Farhadi M., Yousefi R., Chobert J.-M., Haertlé T., Moosavi-Movahedi A.A. (2011). Beta casein-micelle as a nano vehicle for solubility enhancement of curcumin; food industry application. LWT Food Sci. Technol..

[151] Purpura M., Lowery R.P., Wilson J.M., Mannan H., Münch G., Razmovski-Naumovski V. (2018). Analysis of different innovative formulations of curcumin for improved relative oral bioavailability in human subjects. Eur. J. Nutr..

[152] Zheng B., Zhang X., Lin H., McClements D.J. (2019). Loading natural emulsions with nutraceuticals using the ph-driven method: Formation & stability of curcumin-loaded soybean oils bodies. Food Funct..

